# Preferentially expressed genes in stomach adenocarcinoma cells.

**DOI:** 10.1038/bjc.1987.239

**Published:** 1987-11

**Authors:** T. Shiosaka, Y. Tanaka, Y. Kobayashi

**Affiliations:** First Department of Internal Medicine, School of Medicine, Ehime University, Japan.

## Abstract

**Images:**


Br. J. Cancer (1987), 56, 539–544 © The Macmillan Press Ltd., 1987

### Preferentially expressed genes in stomach adenocarcinoma cells

T. Shiosaka, Y. Tanaka & Y. Kobayashi

*First Department of Internal Medicine, School of Medicine, Ehime University, Shigenobu, Onsengun, Ehime, 791-02 Japan.*

**Summary** cDNA clones complementary to mRNA of neoplastic cells of human stomach tissue were used to examine quantitative changes in the mRNA levels of specific genes in neoplastic cells. Poly(A)$^+$RNA from poorly differentiated adenocarcinoma cells of a female patient with stomach cancer was used for construction of a complementary DNA (cDNA) library. Screening of the 18,000 colonies utilizing $^{32}$P-cDNAs derived from normal human tissue and stomach carcinoma tissue samples was used to select clones likely to represent sequences preferentially expressed in stomach carcinoma cells. Twenty-six recombinants were initially selected and further analysis of these clones indicated that eight (4-3D, 9-2D, 9-4G, 29-1A, 29-6F, 37-1B, 115-5A and 52-5F) contain sequences preferentially expressed in stomach carcinoma cells. We have identified the 9-4G, 29-1A, and 29-6F genes which are differentially expressed in human neoplasia.

Cellular commitment to differentiate is the result of inductive and/or repressive alterations in genetic expression. Circumstantial evidence suggests that most cancers are not caused by single genes but are the products of multiple events that probably involve multiple genes (Duesberg, 1976). That process may involve the transcription of genes not previously transcribed. More likely, it involves changes in the abundance of specific mRNAs in the cells. Dermen *et al.* (1981) used cloned cDNA to determine the level at which specific rat hepatoma genes were controlled. Their evidence, using both *in vitro* labelled RNAs from tissue and pulse-labelled cellular RNAs, suggests that the synthesis of most tissue-specific moderately abundant mRNAs is regulated at the level of transcription. By using recombinant DNA technology to prepare a cloned library of expressed gene sequences, Shiosaka and Saunders (1982) have identified several genes that are differentially expressed in human leukaemias.

Nowell (1976) emphasized that a feature of human neoplasia is the proliferative advantage of the neoplastic clone. However, in liver regeneration caused by partial hepatectomy, the number of oncogene transcripts increases concomitantly with the burst of DNA synthesis (Goyette *et al.*, 1983; Fausto & Shank, 1983). One view of the cause of cancer is that it results from an impairment of the cell differentiation process. That process may involve the transcription of genes not previously transcribed. It may also involve the repression of genes normally expressed. It is legitimate to predict that the expression of certain genes might be altered in neoplasia. In this report we describe the preparation of mRNA sequences in the form of a cDNA library whose transcription may specifically occur in neoplastic cells and the use of these sequences to measure the population of mRNA for neoplastic cell specific expressed genes in adenocarcinoma cells from stomach and colon cancer patients.

### Materials and methods

### *Patients*

Stomach and colon specimens were obtained immediately after surgical resection and processed on ice. Tissue samples were taken from tumour as well as the 'normal' area. Ulcerated and necrotic tissue was dissected from the tumour tissue. Thus only intact tumours and normal tissues were used for RNA extraction. Whole thickness slices were taken from the tumour for microscopy. They were fixed in buffered formalin for histological diagnosis.

Correspondence: T. Shiosaka.
Received 26 January 1987; and in revised form, 5 June 1987.

### *Construction of a stomach carcinoma cDNA library*

Total cellular RNA was purified from stomach and colon tissues according to Frazier *et al.* (1981).

For construction of the cDNA library, total RNA was isolated and poly(A)$^+$RNA was selected by oligo d(T) cellulose chromatography essentially as described by Lomedico and Saunders (1977). The construction of the recombinant cDNA library has been described (Efstratiadis *et al.*, 1976). Single stomach cDNA was synthesized with avian reverse transcriptase (Seikagaku Kogyo Inc.) and double stranded cDNA was synthesized with *Escherichia coli* DNA polymerase 1 (Boehringer, Mannheim). After addition of deoxycytidine homopolymer tails, cDNA was cloned into the Pst I site of deoxyguanosine tailed plasmid pBR322. Recombinant plasmid was transformed into *E. coli* K12 (strain RRI) as described by Dagert and Ehrlich (1979). After spreading on L broth/bacto-agar containing tetracycline, 18,000 individual colonies were transferred to microtitre plates (Falcon). The screening was performed essentially as described by Grunstein and Hogness (1976), using $^{32}$P-labelled cDNA ($\sim 1.0 \times 10^6$ cpm ml$^{-1}$) transcripts of poly(A)$^+$RNA extracted from tumour and normal stomach of a gastric carcinoma patient, as probe. Following hybridization, filters were exposed to X-ray films for 1–3 days at $-70$°C in the presence of intensifying screens.

### *Hybridization*

In Northern blot experiments, 30 $\mu$g total RNA was denatured following 1 h incubation at 50°C in 50% DMSO, 1 M glyoxal and 10 mM phosphate buffer pH 7.0 (McMaster & Carmichael, 1977). After electrophoresis through a 1% agarose gel in 10 mM phosphate buffer pH 7.0, RNA was transferred to nitrocellulose (Schleicher & Schuell) and filters were baked for 2 h at 80°C under vacuum (Thomas, 1980). In dot blot hybridization, RNA was heated to 60°C in X6 SSC and 2.2 M formaldehyde for 1 h and chilled on ice. Several dilutions were made in the same solution and 3 $\mu$l aliquots were dotted onto nitrocellulose.

All prehybridization reactions were performed in 50% formamide, X5 SSC, X5 Denhardt's solution and 0.1% SDS at 42°C for 10–15 h. The hybridization buffer was 50% (v/v) formamide, X5 SSC, 50 mM sodium phosphate buffer pH 7.0, X5 Denhardt's solution, 100 $\mu$g ml$^{-1}$ yeast transfer RNA, 100 $\mu$g ml$^{-1}$ poly(A), 0.1% SDS and $^{32}$P-labelled probe at 42°C for 15 h. After hybridization filters were washed with 3 changes of X2 SSC and 0.1% SDS for 5 min each at room temperature with 3 changes of X0.2 SSC and 0.1% SDS for 5 min each at room temperature and with 3 changes of X0.16 SSC and 0.1% SDS for 15 min each at 50°C. The filters were exposed to X-ray film at $-70$°C using a Fuji intensifying screen (Hi-screen). Scanning densitometry of autoradiographic films was performed using a Digital Densitometer of TOYO Model DMN-33C.

*DNA sequencing*

The inserted fragments of 9-4G, 29-1A and 29-6F cDNA clones were cloned into the M13 vectors mp18 and mp19 and were sequenced by the dideoxy chain termination technique (Sanger *et al.*, 1977; Vieira & Messing, 1982).

### Results

*Isolation and initial selection of recombinant clones*

The library was screened with [$^{32}$P]-cDNA synthesized to high specific activity from poly(A)$^+$RNA of tumour and normal stomach tissue of the stomach cancer patient. Out of 18,000 initial colonies, 67% displayed a hybridization signal more intense than controlled colonies containing plasmid pBR322 without a cDNA insert. Clones containing cDNA transcripts of rare mRNAs and very short cDNA inserts were not expected to react in the colony hybridization experiments. Twenty-six clones that reacted positively with cDNA transcripts of poly(A)$^+$RNA from the tumour tissue and did not react with those from normal stomach tissue were selected and used in subsequent RNA titration experiments. For more quantitative studies on the expression of selected clones, titration with a RNA dot hybridization analysis was used to estimate the relative abundances of RNA to these 26 clones in the RNAs from normal and tumour tissue. The intensity of the observed spots was compared by scanning densitometry of the films and the results are shown in Figure 1. Sequences 4-3D, 9-2D, 9-4G, 29-1A, 29-6F, 37-1B, 52-5F and 115-5A compared to 42-5B and 63-3D were preferentially expressed in tumour tissue (Figures 1 and 2). No hybridization of the clones (9-2D, 9-4G, and 115-5A) was detectable with filters containing 0.03–0.5 $\mu$g of poly(A)$^+$RNA from the normal stomach tissue of the cancer specimen after a 3 day exposure and a very faint signal could be scanned with clone 4-3D, 29-1A, 29-6F, 37-1B and 52-5F (Figures 1 and 2). To ensure that the observed differences were due to specific hybridization of our probes with a discrete RNA species and to determine the size of the corresponding mRNA, the Northern blot hybridization technique was adopted. The pictures of 9-4G and 29-1A are shown in Figures 3 and 4 which is a Northern blot of total RNA from one normal stomach tissue area, one adenocarcinoma from the same individual, one adenocarcinoma from the stomach cancer, two adenocarcinomas from colon cancer, one pancreatic cancer and one hepatoma. The histological diagnosis of these tumours is given in Table I. The intensity of the observed bands was compared by scanning densitometry of the films and integration of the peak areas, and the results are given in Figures 5 and 6. It is evident that the increase of 29-1A and 9-4G mRNA in tumour RNA is remarkable. These results were confirmed in at least two different Northern blots. The differential representation of 9-2D, 9-4G, 29-1A and 29-6F clone with various kinds of mRNA from normal stomach and colon and adenocarcinoma cells was examined repeatedly and is summarized in Table II. No hybridization of 9-4G and 29-6F was detectable with filters containing 30 $\mu$g total RNA from normal tissue after 3 days of exposure.

*Sequence analysis of the inserted fragments of 9-4G, 29-1A and 29-6F cDNA clones*

The nucleotide sequences of inserted fragments of 9-4G, 29-1A and 29-6F cDNA clones were subcloned into M13 mp

**Table I** Histology of carcinomas

| | Tumour histology |
|---|---|
| Gastric cancer 1 | Mucinous adenocarcinoma |
| Gastric cancer 2 | Moderately differentiated adenocarcinoma |
| Colon cancer 1 | Well differentiated tubular adenocarcinoma |
| Colon cancer 2 | Well differentiated adenocarcinoma |
| Metastatic breast cancer | Well differentiated adenocarcinoma |
| Pancreas cancer | Adenocarcinoma |
| Hepatoma | Hepatocellular carcinoma |

**Figure 1** Relative level of selected clone mRNA in normal stomach tissue and carcinoma from stomach cancer patient. 30–500 ng poly(A)$^+$RNA were spotted on to nitrocellulose and hybridized to each $^{32}$P-labelled clone. The picture of dot blot hybridization to 9-2D, 9-4G, 29-1A, 29-6F, 37-1B, 52-5F, 115-5A, 42-5B and 63-3D clones is shown in **Figure 2**. The intensity of the spots observed in Northern blot hybridizations was determined by densitometry of the films. The values are expressed in arbitrary units. Solid bar; poly(A)$^+$RNA from stomach tumour tissue, Open bar; poly(A)$^+$RNA from normal stomach tissue.

**Figure 2** Dot blot hybridization analysis of selected clone mRNA in normal stomach area and carcinoma. 500 ng poly(A)$^+$RNA and serial dilutions were spotted on to nitrocellulose and hybridized to each $^{32}$P-labelled clone. C; poly(A)$^+$RNA from stomach tumour tissue, N; poly(A)$^+$RNA from normal tissue.

**Figure 3** Northern blot analysis of 9-4G mRNA in normal stomach tissue, two stomach carcinomas, two colon carcinomas, a pancreatic carcinoma and a hepatoma. Total RNA was isolated; and 30 $\mu$g samples were electrophoresed in each lane of a 1% agarose gel and subsequently transferred to nitrocellulose as described by Thomas (1980). Hybridization was carried out with clone 9-4G. The source of RNAs in the various lanes was as follows: 1, normal stomach tissue; 2, stomach tumour tissue 1; 3, stomach tumour tissue 2; 4, colorectal cancer tissue 1; 5, colorectal cancer tissue 2; 6, metastatic breast cancer tissue; 7, pancreatic cancer tissue; 8, hepatoma. The histology of these tissues is given in **Table I**.

**Figure 4** Northern blot analysis of 29-1A mRNA in normal stomach tissue, 2 stomach carcinomas, 2 colorectal carcinomas, one pancreas carcinoma and one hepatoma. Total RNA was prepared from each normal and tumour tissue; 30 $\mu$g was used in each lane. Nick translation of the 29-1A prove and hybridization conditions were as in **Figure 3**. For sources of RNAs in the various lanes see **Figure 3** and for histology of the extracted tissues see **Table I**.

**Figure 5** Relative levels of 9-4G mRNA in normal stomach tissue, 2 stomach carcinomas, 2 colon carcinomas, one pancreas carcinoma and one hepatoma. The intensity of the bands observed in Northern blot hybridizations was determined by scanning densitometry of the films. The values obtained after integration of the peak areas, expressed in arbitrary units, are given in the histogram. For sources of RNAs in the various lanes see **Figure 3** and for histology of the extracted tissues see **Table I**.

**Figure 6** Relative levels of 29-1A mRNA in normal stomach tissue (as shown in **Figure 4**), 2 stomach carcinomas, 2 colon carcinomas, one pancreatic carcinoma and one hepatoma. The conditions are as for **Figure 4**. For sources of RNAs in the various lanes see **Figure 3** and for histology of the extracted tissues see **Table I**.

```
9-4G

        10        20        30        40        50        60        70        80
AACCTTTGAATTTAATCCATTTACATTCAAGGTTATTATTTATAGGTGAAGTCTTACTCCTGTTATTTTGGTTAATTGTT

        90       100       110       120       130       140       150       160
TTATTGTAGTTTTGTATGTTCTTTGTTCTTTTCCTCTCTTATTGTTTAGTATTGTGGTTTGATGATTTTCTGTAGTATAA

       170       180

AAGTTTGATTATA

29-1A

        10        20        30        40        50        60        70        80
ATCCTAGTAGGTTGTAAGCATCCAAGAAATAATCATTTCCTCTTAGTTTTTTTACTTGTTAGCATATATTTGTTCATAGT

        90       100       110       120       130       140       150       160
AATTTCTACTGATTGCCACTTCTACAGTATCAGTGTAACTGTCTCCTTTTTCATTTTTTATTTTATTTATTAGAGTTTTC

       170       180       190       200       210       220       230

TTTCTTTTTTCTTAGTCTAGATAATGGTTTGCCAATTGTATTTATCTAGACTAAAAAAAAAAAAAAAAA

29-6F

        10        20        30        40        50        60        70        80
ACCTCACTACAGAAGATATTCAGATGGCAAATGAACATATGAAAATGCTCAACATCTTACATCATAGGAAATTACAAATT

        90       100       110       120       130       140       150       160
ATAATAATTGGGAGCCCCAATGTTGGGTGCAGAGATACTTAAAATTGTTATATCCTTTTGCTGAATTGACTCCTTTATCA

       170       180       190       200       210       220       230       240
TTATATAAGTGACCTTTCTTTGTCTTCTTTTACAGTCATTGATTTATGTCTATTTTATCTAAATATAGCTACTCTTGCTC

       250       260       270       280       290       300       310       320
TTTTTGACCTCCAGTTGTATGGAATGTGTTTTCCCATCCCTCACTCTCAGTCTATGTGTGTCTTTATAGGTGAGTGGGTT

       330       340       350       360       370       380       390       400
TCTTCGTAGGCTGCATATAGTTGAGTCTTGTTTCTTTATCAAGCCTCTCGCTGCCTTTTAATTGGAAAATTGAGACCCTT

       410       420       430       440       450       460       470       480
TACATTCAGTATTATATTGATAAGCAAGGACTTACTACTGCCATCTTGTTGCTTATTTCTGGTTTTGTAACTTTTGTCTT

       490       500       510       520       530       540       550       560

CCTTTCCCCCCCCCCCCTCCAGCCTGGGCAACAGAATGAAACTTTGTCTCAAAAAAAAAAAAAAAAAAATCATCTGAAGG

       570       580       590       600       610       620       630       640
TACAAAACTCTCACTGGTTACAGTAAGTACACGGAAGAACAGAATATTAAACATTGTATGTGTGGTGTGTAAACTACTCT

       650       660       670       680       690       700       710       720

TAAGTGGAAAGACTAAATGATGAACTAATCAAAAATAATAACCACAACAACTTTTCAAGACCAAGTCAGTACCATAAGAT

       730
AAATAAAAA
```

**Figure 7** cDNA sequences of selected clones (9-4G, 29-1A and 29-6F) by dideoxy chain termination technique.

vectors and determined. Figure 7 shows 173 nucleotides of 9-4G, 229 nucleotides of 29-1A and 729 nucleotides of 29-6F clones. The sequences of these cDNA clones (9-4G, 29-1A and 29-6F) are not present in those of the data base of GenBank recorded up to 1987.

### Discussion

Phenotypic differences between cells arise from qualitative and quantitative differences in protein expression, and these in turn reflect differences in mRNA composition of the cells. For example, changes in the relative abundance of mRNAs occurring during normal differentiation of cells or as a consequence of neoplastic transformation have been demonstrated (Chikaraishi *et al.*, 1978; Caplan & Ordahl, 1978; Wald *et al.*, 1978; Moyzis *et al.*, 1980). Previously, we have shown that the application of recombinant DNA technology makes it possible to analyze very sensitively the difference in gene expression between normal and neoplastic cells. cDNA clones complementary to mRNA of cells from patients having chronic lymphocytic leukaemia were used to examine quantitative changes in the mRNA levels of specific genes in human leukaemias. This approach could be important for the subclassification of leukaemias (Shiosaka & Saunders, 1982).

In this experiment, we have constructed a library of 18,000 sequences, which would statistically represent 67% of the more abundant mRNAs present in the tumour. Clearly, sequences which are present at very low abundance may not be represented in such a small library. However, our aims were to identify mRNAs expressed in the cancer and not

**Table II** Relative expression of 9-2D, 9-4G, 29-1A and 29-6F poly(A)$^+$RNA in normal and cancer tissues

| | | *Relative expression of poly(A)$^+$RNA* | | | |
|---|---|---|---|---|---|
| | *Tumour histology* | *9-2D* | *9-4G* | *29-1A* | *29-6F* |
| Normal | | | | | |
| Gastric tissue | | + | − | − | − |
| Gastric tissue | | + | − | − | − |
| Colon tissue | | − | − | − | − |
| Colon tissue | | − | − | − | − |
| Cancer | | | | | |
| Gastric cancer | Mucinous ad. | +++ | +++ | ± | + |
| Gastric cancer | Moderatory diff. ad. | ND | +++ | +++ | +++ |
| Gastric cancer | Poorly diff. ad. | +++ | +++ | − | +++ |
| Gastric cancer | Poorly diff. ad. | + | ± | + | ± |
| Colon cancer | Well diff. ad. | ++ | − | ± | ++ |
| Colon cancer | Well diff. tubular ad. | +++ | ++ | + | ND |
| Colon cancer | Well diff. ad. | +++ | +++ | +++ | +++ |
| Colon cancer | Well diff. ad. | − | + | ± | + |
| Colon cancer | Well diff. ad. | ++ | − | + | +++ |

The intensity of bands observed on Northern blot hybridization was determined by scanning densitometry of the films and calculated as rate of maximal value. To compare the results obtained in different experiments at least one RNA sample was included in each blot as a reference. Each sample was analyzed in at least 2 different Northern blots. The relative expression of each clone poly(A)$^+$RNA was scored as follows: − negative (0%), ± weak (1–20%), + positive (20–40%), ++ moderately (40–60%), +++ strong (60–100%). Diff., differentiated, ad., adenocarcinoma. ND, not done.

expressed in the normal stomach tissue, and to examine their changing patterns of transcription in dysplasia. In the experiments presented here, only three genes (9-4G, 29-1A and 29-6F) that are differentially expressed in human adenocarcinoma cells were identified. We observed almost no hybridization of these probes with mRNAs from normal stomach and colon tissue. These selected genes were not found to be amplified in the stomach and colon tumour DNA (unpublished data). When DNAs from stomach cancer and normal stomach tissue were examined by Southern hybridization using these selected clones, no appreciable restriction fragment length polymorphism was observed (unpublished data). However, as these selected genes represent one part of each cDNA, it is not always possible to find DNA polymorphism in stomach cancer DNA using these selected clones.

Control of gene expression at the level of transcription has been demonstrated for haemoglobin (Groudine & Weintraub, 1980), fibronectin, collagen (Tyagi *et al.*, 1983), c-*myc* and c-*fos* (Greenberg & Ziff, 1984; Kelly *et al.*, 1983; Cochran *et al.*, 1984) and other proteins. Transcriptional level control is not universal, however. Significant regulation of RNA at the level of RNA processing or degradation has been shown for a variety of growth regulated gene products including c-*myc* (Dani *et al.*, 1984) and dihydrofolate reductase (Leys *et al.*, 1984). The cause of the high concentration of 9-4G, 29-1A and 29-6F mRNAs which are expressed in adenocarcinoma are as yet unknown. However, such differences might provide an additional marker of an entirely new kind that could be useful in cancer diagnosis.

Clones 9-4G, 29-1A and 29-6F hybridized to RNA of a different size. Expression of cellular homologues of viral oncogenes has been observed in a variety of neoplastic cell types (Hayward *et al.*, 1981; Schwab *et al.*, 1983; Slamon *et al.*, 1984; Calabretta *et al.*, 1985). Comparison of the DNA sequences of these clones (9-4G, 29-1A and 29-6F) and viral oncogenes, which had been reported, reveals that these selected clones are not related to viral oncogenes. At this stage it is too early to say whether the sequences which we have defined represent important mRNAs whose levels of transcription accurately reflect changes in transformation of cells. However, it is important to understand why transcription of these sequences is so remarkably increased in stomach and colon cancer, and especially to investigate the structure of promoter against these sequences on neoplastic tissue DNA. Our findings demonstrate that the construction and comparative screening of a cDNA library is a powerful approach in the identification of cancer markers for the diagnosis and prospective evaluation of many types of malignancy.

This study was supported in part by a grant-in-aid for scientific research from the Ministry of Education, Science and Culture of Japan (no. 60570293).

### References

CALABRETTA, B., KCZMAREK, L., MING, P.M.L., AU, F. & MING, S.C. (1985). Expression of c-myc and other cell cycle-dependent genes in human clone neoplasia. *Cancer Res.*, **45**, 6000.

CAPLAN, A.I. & ORDAHL, C.P. (1978). Irreversible gene repression model for control of development. *Science*, **201**, 120.

CHIKARAISHI, D.M., DEEB, S.S. & SUEOKA, N. (1978). Sequence complexity of nuclear RNAs in adult rat tissues. *Cell*, **13**, 111.

COCHRAN, B.H., ZULLO, J., VERNA, I.M. & STILES, C.D. (1984). Expression of the c-fos gene and of an fos-related gene is stimulated by platelet-derived growth factor. *Science*, **226**, 1080.

DAGERT, M. & EHRILICH, S.D. (1979). Prolonged incubation in calcium chloride improves the competence of *Escherichia coli* cells. *Gene*, **6**, 23.

DANI, C., BLANCHARD, J.M., PIECHACZYK, M., EL SABATONY, S., MARTY, L. & JEANTEUR, P. (1984). Extreme instability of myc mRNA in normal and transformed human cells. *Proc. Natl Acad. Sci. USA*, **81**, 7046.

DERMAN, E., KRAUSTER, K., WALLING, L., WEINBERGER, C., RAY, M. & DARNELL, J.E. (1981). Transcriptional control in the production of liver-specific mRNAs. *Cell*, **23**, 731.

DUESBERG, P.H. (1976). Activated proto-onc genes: Sufficient or necessary for cancer? *Science*, **228**, 669.

EFSTRATIADIS, A., KAFATOS, F.C., MAXAM, A.M. & MANIATIS, T. (1976). Enzymatic *in vitro* synthesis of globin genes. *Cell*, **7**, 279.

FAUSTO, N. & SHANK, P.R. (1983). Oncogene expression in liver regeneration and hepatocarcinogenesis. *Hepatology*, **3**, 1016.

FRAZIER, M.L., MONTAGNA, R.A. & SAUNDERS, G.F. (1981). Insulin gene expression during development of the fetal bovine pancreas. *Biochemistry*, **20**, 367.

GOYETTE, M., PETROPOULOS, C.J., SHANK, P.R. & FAUSTO, N. (1983). Expression of a cellular oncogene during liver regeneration. *Science*, **219**, 510.

GREENBERG, M.E. & ZIFF, E.B. (1984). Stimulation of 3T3 cells induces transcription of the c-fos proto-oncogene. *Nature*, **311**, 433.

GROUDINE, M. & WEINTRAUB, H. (1980). Activation of cellular genes by avian RNA tumor viruses. *Proc. Natl Acad. Sci. USA*, **77**, 5351.

GRUNSTEIN, M. & HOGNESS, D.S. (1976). A method for the isolation of cloned DNAs that contain a specific gene. *Proc. Natl Acad. Sci. USA*, **72**, 3961.

HAYWARD, W.S., NEEL, B.G. & ASTRIN, S.M. (1981). Activation of a cellular onc gene by promoter insertion in ALV-induced lymphoid leukosis. *Nature*, **290**, 475.

KELLY, K., COCHRAN, B.H., STILES, C.D. & LEDER, P. (1983). Cell-specific regulation of the c-myc gene by lymphocyte mitogens and platelet-derived growth factor. *Cell*, **35**, 603.

LEYS, E.J., CROUSE, G.F. & KELLEMS, R.E. (1984). Dihydrofolate reductase gene expression in cultured mouse cell is regulated by transcript stabilization in the nucleus. *J. Cell Biol.*, **99**, 180.

LOMEDICO, P.T. & SAUNDERS, G.F. (1977). Cell-free modulation of proinsulin synthesis. *Science*, **198**, 620.

McMASTER, G.K. & CARMICHAEL, G.G. (1977). Analysis of single- and double-stranded nucleic acids on polyacrylamide and agarose gels by using glyoxal and acridine orange. *Proc. Natl Acad. Sci. USA*, **74**, 4835.

MOYZIS, R.K., GRANDY, D.L., LI, D.W., MIRRIS, S.E. & TS'O, P.O. (1980). Extensive homology of nuclear ribonucleic acid and polysomal poly(adenylic acid) messenger ribonucleic acid between normal and neoplastically transformed cell. *Biochemistry*, **19**, 821.

NOWELL, P.C. (1976). The clonal evolution of tumor cell populations. *Science*, **174**, 23.

SANGER, F., NICKLEN, S. & COULSON, A. (1977). DNA sequence with chain terminating inhibitors. *Proc. Natl Acad. Sci. USA*, **74**, 5463.

SCHWAB, M., ALITALO, K., VARMUS, H.E., BISHOP, J.M. & GEORGE, D. (1983). A cellular oncogene (c-Ki-*ras*) is amplified, overexpressed, and located within karyotypic abnormalities mouse adenocortical tumor cells. *Nature*, **303**, 497.

SHIOSAKA, T. & SAUNDERS, G.F. (1982). Differential expression of selected genes in human leukemia leukocytes. *Proc. Natl Acad. Sci. USA*, **79**, 4668.

SLAMON, D.J., DEKERNION, J.B., VERMA, I.M. & CLINE, M.J. (1984). Expression of cellular oncogene in human malignancies. *Science*, **224**, 256.

THOMAS, P.S. (1980). Hybridization of denatured RNA and small DNA fragments transferred to nitrocellulose. *Proc. Natl Acad. Sci. USA*, **77**, 5201.

TYAGI, J.S., HIRANO, M., MERLINO, G.T. & PASTAN, I. (1983). Transcriptional control of the fibronectin gene in chick embryo fibroblasts transformed by Rous Sarcoma virus. *J. Biol. Chem.*, **258**, 5783.

VIEIRA, J. L MESSING, J. (1982). The pUC plasmids, an M13mp7-derived system for insertion mutagenesis and sequencing with synthetic universal primers. *Gene*, **19**, 259.

WALD, B.J., KLEIN, W.H., HOUGH-EVANS, B.R., BRITTEN, R.J. & DAVIDSON, E.H. (1978). Sea urchin embryo mRNA sequences expressed in the nuclear RNA of adult tissues. *Cell*, **14**, 941.


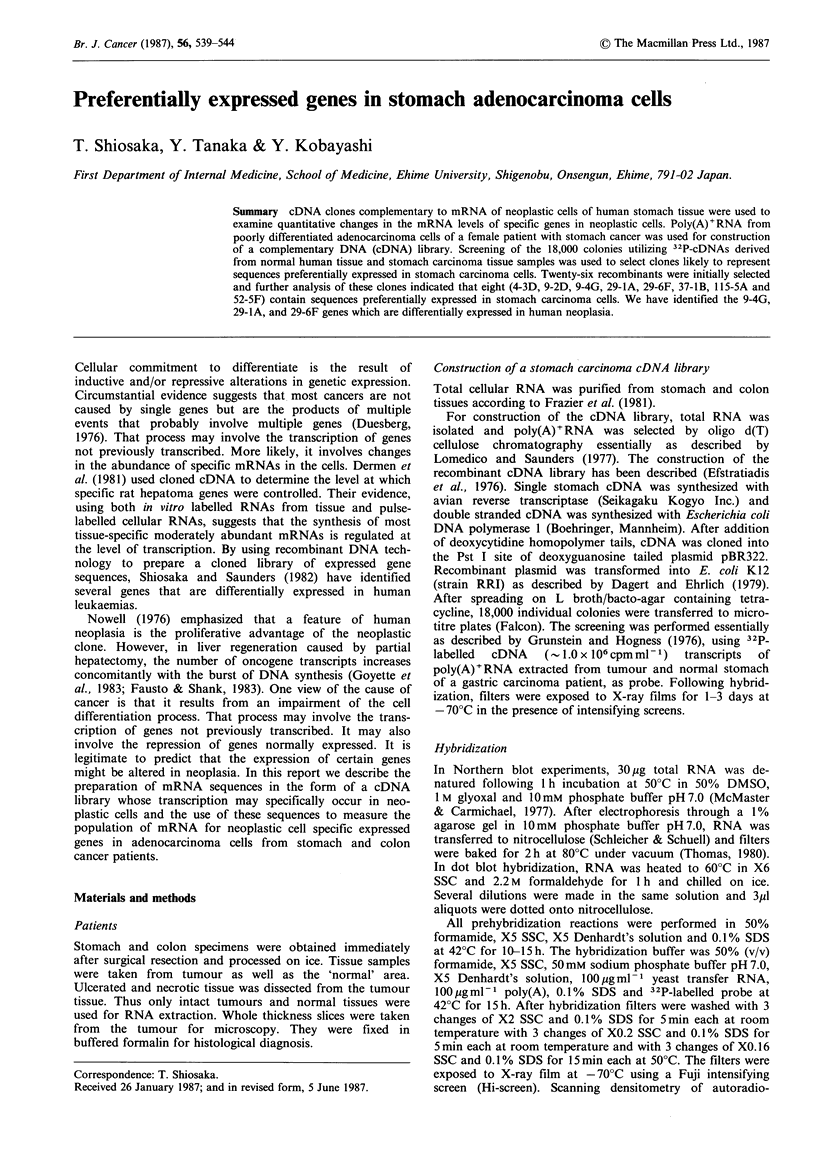

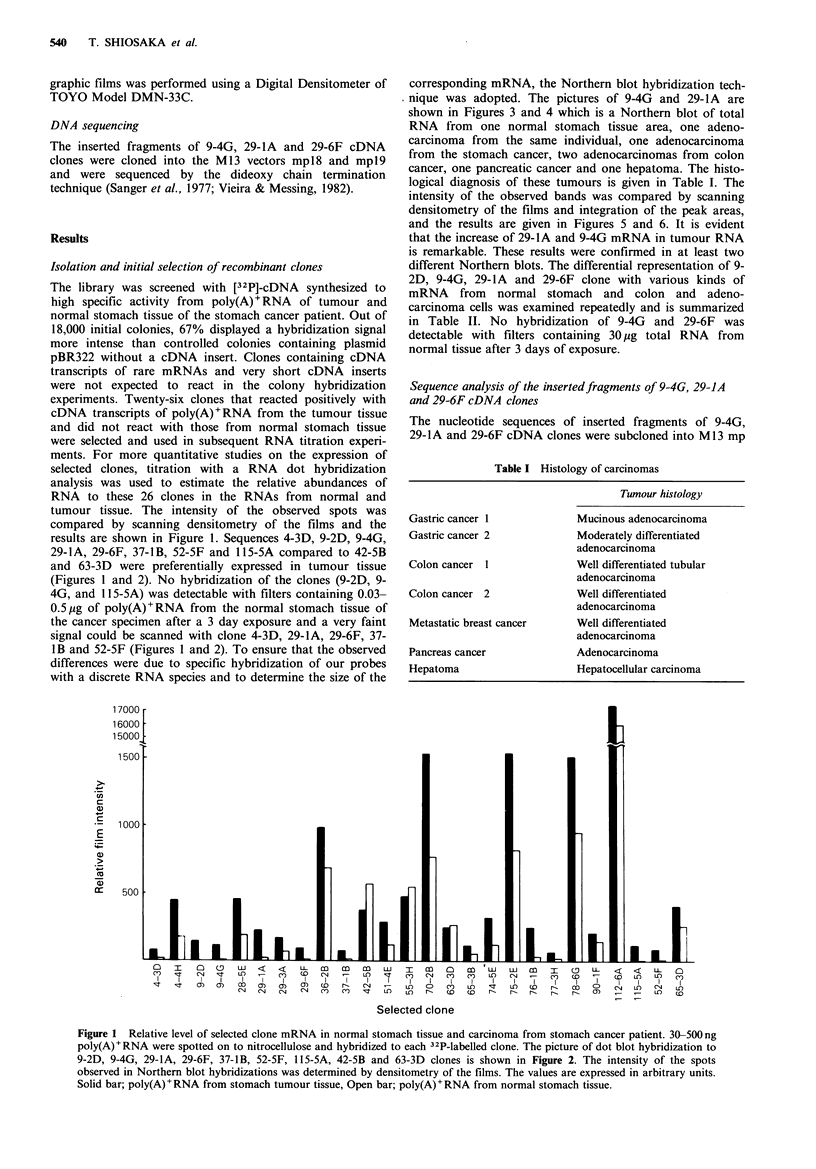

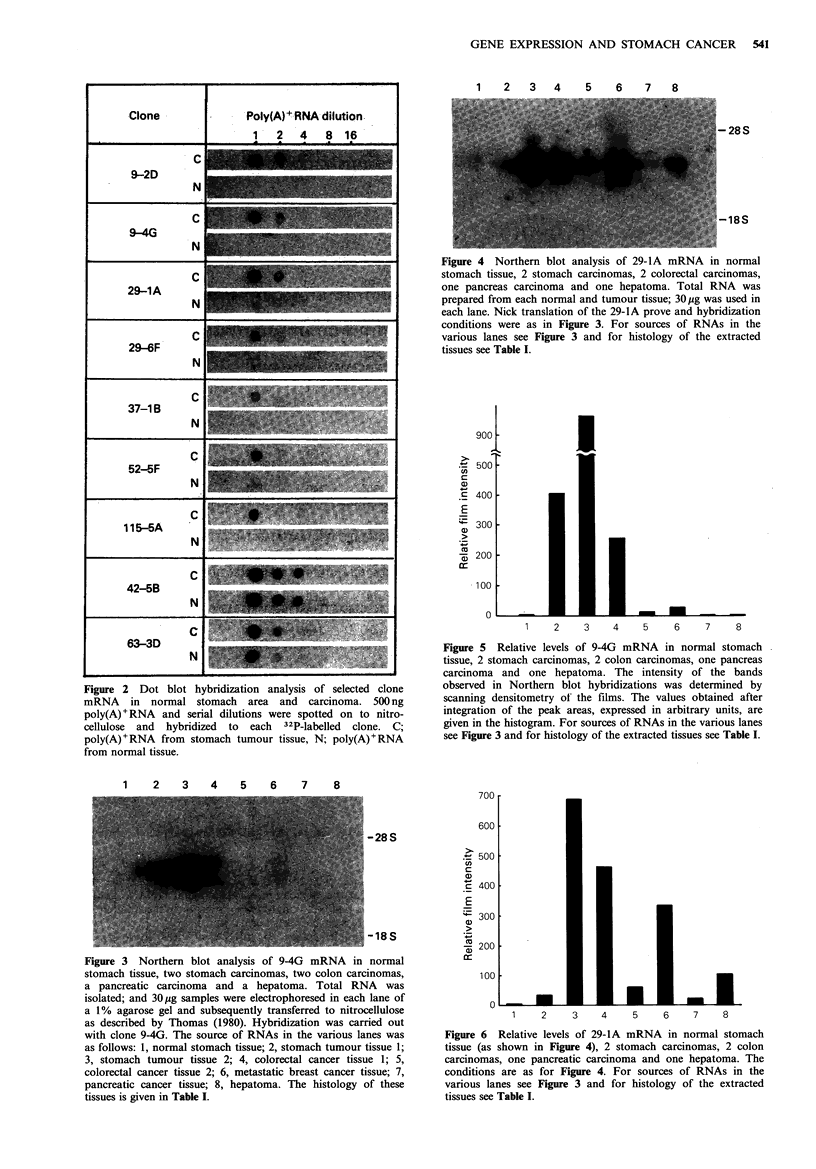

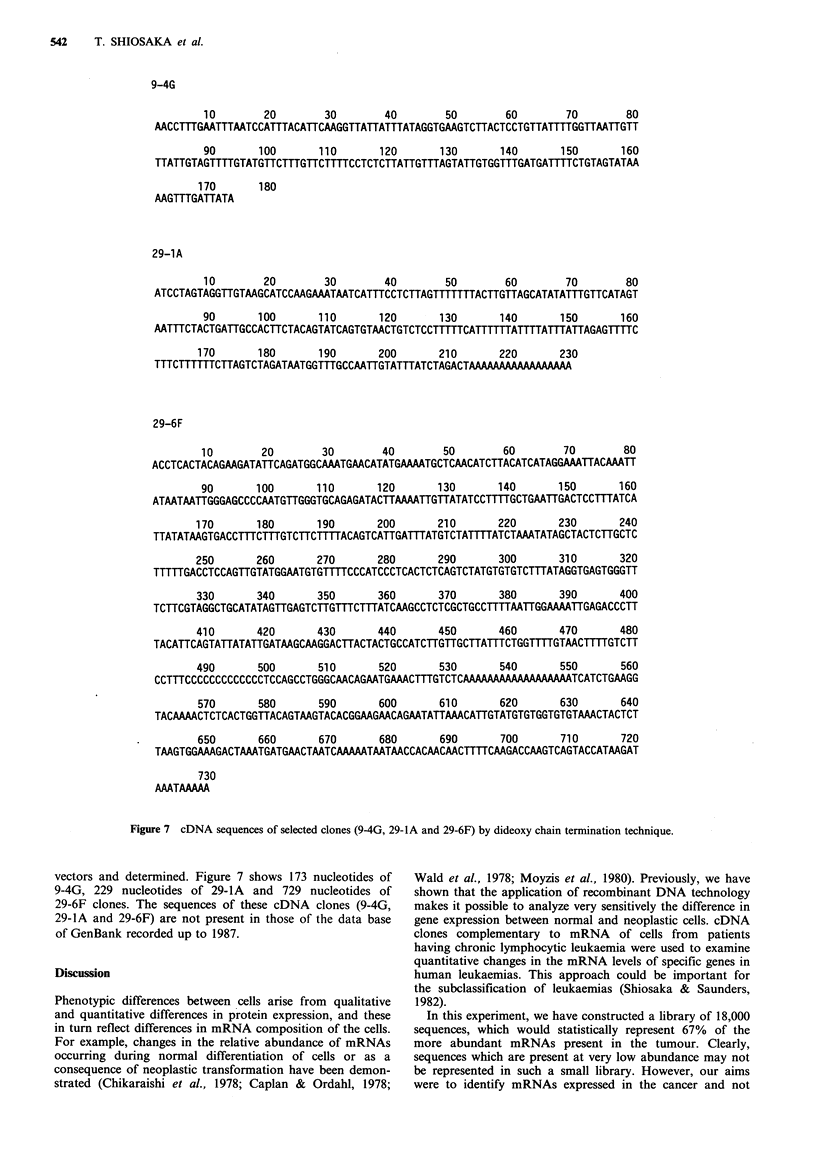

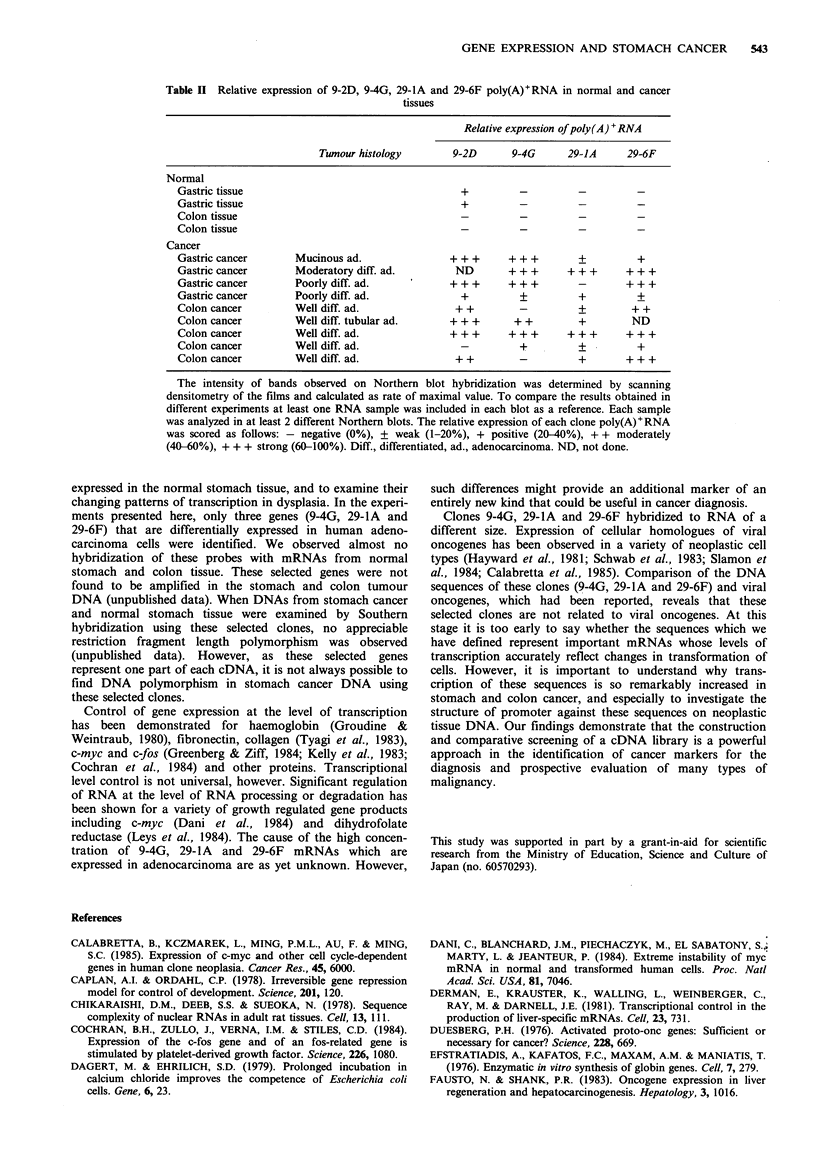

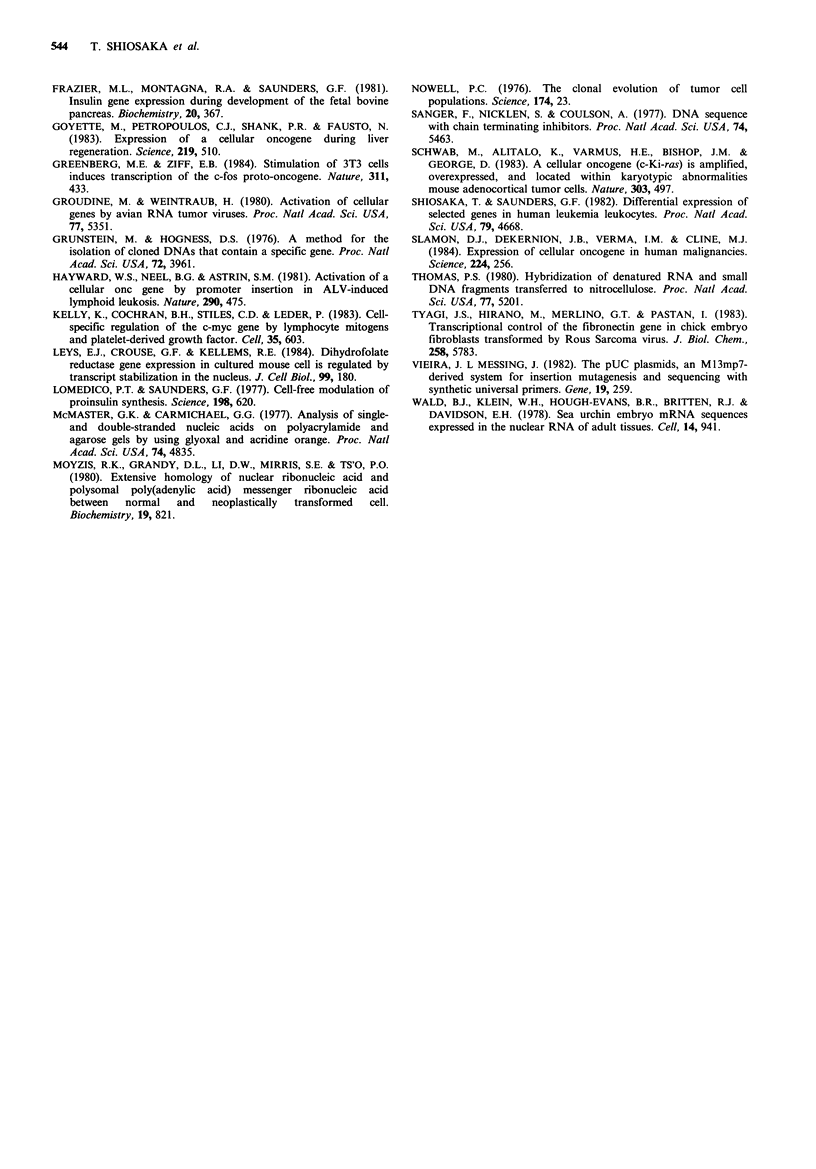


## References

[OCR_00597] Calabretta B., Kaczmarek L., Ming P. M., Au F., Ming S. C. (1985). Expression of c-myc and other cell cycle-dependent genes in human colon neoplasia.. Cancer Res.

[OCR_00602] Caplan A. I., Ordahl C. P. (1978). Irreversible gene repression model for control of development.. Science.

[OCR_00606] Chikaraishi D. M., Deeb S. S., Sueoka N. (1978). Sequence complexity of nuclear RNAs in adult rat tissues.. Cell.

[OCR_00610] Cochran B. H., Zullo J., Verma I. M., Stiles C. D. (1984). Expression of the c-fos gene and of an fos-related gene is stimulated by platelet-derived growth factor.. Science.

[OCR_00615] Dagert M., Ehrlich S. D. (1979). Prolonged incubation in calcium chloride improves the competence of Escherichia coli cells.. Gene.

[OCR_00622] Dani C., Blanchard J. M., Piechaczyk M., El Sabouty S., Marty L., Jeanteur P. (1984). Extreme instability of myc mRNA in normal and transformed human cells.. Proc Natl Acad Sci U S A.

[OCR_00626] Derman E., Krauter K., Walling L., Weinberger C., Ray M., Darnell J. E. (1981). Transcriptional control in the production of liver-specific mRNAs.. Cell.

[OCR_00631] Duesberg P. H. (1985). Activated proto-onc genes: sufficient or necessary for cancer?. Science.

[OCR_00635] Efstratiadis A., Kafatos F. C., Maxam A. M., Maniatis T. (1976). Enzymatic in vitro synthesis of globin genes.. Cell.

[OCR_00639] Fausto N., Shank P. R. (1983). Oncogene expression in liver regeneration and hepatocarcinogenesis.. Hepatology.

[OCR_00645] Frazier M. L., Montagna R. A., Saunders G. F. (1981). Insulin gene expression during development of the fetal bovine pancreas.. Biochemistry.

[OCR_00650] Goyette M., Petropoulos C. J., Shank P. R., Fausto N. (1983). Expression of a cellular oncogene during liver regeneration.. Science.

[OCR_00655] Greenberg M. E., Ziff E. B. (1984). Stimulation of 3T3 cells induces transcription of the c-fos proto-oncogene.. Nature.

[OCR_00660] Groudine M., Weintraub H. (1980). Activation of cellular genes by avian RNA tumor viruses.. Proc Natl Acad Sci U S A.

[OCR_00665] Grunstein M., Hogness D. S. (1975). Colony hybridization: a method for the isolation of cloned DNAs that contain a specific gene.. Proc Natl Acad Sci U S A.

[OCR_00670] Hayward W. S., Neel B. G., Astrin S. M. (1981). Activation of a cellular onc gene by promoter insertion in ALV-induced lymphoid leukosis.. Nature.

[OCR_00675] Kelly K., Cochran B. H., Stiles C. D., Leder P. (1983). Cell-specific regulation of the c-myc gene by lymphocyte mitogens and platelet-derived growth factor.. Cell.

[OCR_00680] Leys E. J., Crouse G. F., Kellems R. E. (1984). Dihydrofolate reductase gene expression in cultured mouse cells is regulated by transcript stabilization in the nucleus.. J Cell Biol.

[OCR_00685] Lomedico P. T., Saunders G. F. (1977). Cell-free modulation of proinsulin synthesis.. Science.

[OCR_00689] McMaster G. K., Carmichael G. G. (1977). Analysis of single- and double-stranded nucleic acids on polyacrylamide and agarose gels by using glyoxal and acridine orange.. Proc Natl Acad Sci U S A.

[OCR_00695] Moyzis R. K., Grady D. L., Li D. W., Mirvis S. E., Ts'o P. O. (1980). Extensive homology of nuclear ribonucleic acid and polysomal poly(adenylic acid) messenger ribonucleic acid between normal and neoplastically transformed cells.. Biochemistry.

[OCR_00702] Nowell P. C. (1976). The clonal evolution of tumor cell populations.. Science.

[OCR_00706] Sanger F., Nicklen S., Coulson A. R. (1977). DNA sequencing with chain-terminating inhibitors.. Proc Natl Acad Sci U S A.

[OCR_00711] Schwab M., Alitalo K., Varmus H. E., Bishop J. M., George D. (1983). A cellular oncogene (c-Ki-ras) is amplified, overexpressed, and located within karyotypic abnormalities in mouse adrenocortical tumour cells.. Nature.

[OCR_00717] Shiosaka T., Saunders G. F. (1982). Differential expression of selected genes in human leukemia leukocytes.. Proc Natl Acad Sci U S A.

[OCR_00722] Slamon D. J., deKernion J. B., Verma I. M., Cline M. J. (1984). Expression of cellular oncogenes in human malignancies.. Science.

[OCR_00727] Thomas P. S. (1980). Hybridization of denatured RNA and small DNA fragments transferred to nitrocellulose.. Proc Natl Acad Sci U S A.

[OCR_00738] Vieira J., Messing J. (1982). The pUC plasmids, an M13mp7-derived system for insertion mutagenesis and sequencing with synthetic universal primers.. Gene.

[OCR_00743] Wold B. J., Klein W. H., Hough-Evans B. R., Britten R. J., Davidson E. H. (1978). Sea urchin embryo mRNA sequences expressed in the nuclear RNA of adult tissues.. Cell.

